# Sex Workers’ Work-Related Victimisation and Drug Use During the First Year of the COVID-19 Pandemic in Switzerland

**DOI:** 10.1007/s43576-022-00045-2

**Published:** 2022-02-08

**Authors:** Lorena Molnar, Jenny Ros

**Affiliations:** 1 School of Criminal Sciences, University of Lausanne, Quartier Unil-Sorge, Batiment Batochime, Office 6903, Lausanne, Switzerland; 2 Faculty of Social Work (HETSL | HES-SO), University of Applied Science and Arts Western Switzerland, Lausanne, Switzerland

**Keywords:** Prostitution, Observation, Questionnaire, Difficult-to-reach population

## Abstract

Criminologists have monitored the coronavirus pandemic’s effects on crime and criminal justice since the pandemic’s outbreak. Nonetheless, vulnerable and difficult-to-reach populations have been understudied thus far. This study sheds light on the experiences of sex workers (SW) during the first year of the coronavirus in Switzerland, a country where prostitution is legal. Based upon 40 questionnaires with SW outdoors and indoors and 50 h of field observation, SW reported that the pandemic has had adverse financial and psychosocial effects on them. During the first year of COVID-19, seventeen SW were victims of at least one work-related offence, the most prevalent of which were theft and fraud. Nevertheless, most SW did not report the incidents to the police. Comparing the non-victims with victims, we found that victims, particularly those of multiple crimes, are younger, more often foreigners from extra-EU countries, in an illegal situation and needed to work face to face during the prostitution ban during the lockdown in Switzerland. However, despite these circumstances, most SW do not use illegal drugs, and only a few of them used more during the pandemic. Our research findings were similar to those reported in former studies, although we could infer that the violent victimisation of our sample is less and none of the SW indicated violence on the part of the police. Nevertheless, we have no point of comparison with former years and thus propose a periodic crime victim survey of SW, as well as further prevention measures in the prostitution area.

## Introduction

This article adopts a criminological perspective and addresses the experiences of sex workers (SW) during the first year of the coronavirus pandemic in Switzerland, one of the few countries in the world where sex work is legal (Danna, 2014) and also one in which the coronavirus-related lockdown was rather flexible. Notably, Switzerland is a central European nation of small size (roughly 8.5 million inhabitants) that imposed only a facultative lockdown for its population from the spring until the summer of 2020. Nevertheless, the country also declared a mandatory lockdown for several months for many “non-essential” businesses, amongst which was prostitution (*SR 818.101.24 Ordinance 3 of 19 June 2020 on Measures to Combat the Coronavirus (COVID-19) (COVID-19 Ordinance 3))*.

Since its outbreak, the pandemic has had serious adverse effects on societies and individuals, i.e. the worldwide economy has been affected seriously, people’s mental health has worsened, and their alcohol and drug consumption have increased (Cohut, 2021); further, crime declined in the streets (Nivette et al., 2021), but increased in cyberspace (Buil-Gil et al., 2021). However, other crimes, such as intimate partner violence, increased considerably during the lockdown (see Piquero et al., 2021), although intimate partner homicides did not follow the same trend and remained stable or even declined (Aebi et al., 2021; Asik & Nas Ozen, 2021; Hoehn-Velasco et al., 2021). As expected, criminologists have been studying and monitoring many crime trends during the pandemic, except those suffered by vulnerable and excluded groups, such as SW. Although scholars have made comments and calls for action (see e.g. Howard, 2020; Kawala et al., 2020; Platt et al., 2020; Singer et al., 2020), few empirical studies have been published to date vis-à-vis SW’ experiences related to violence during the pandemic. Nonetheless, this is understandable: To interview or survey SW during a lockdown is highly challenging because of the prohibition to be on the streets and the respondents and researchers’ risk of contagion (Silva & Câmara, 2020), as well as the clandestinisation of sex work during the epidemic (Azam et al., 2021; Callander et al., 2021), all of which have rendered SW even more difficult to reach than usual. Nevertheless, it has been found in several countries that both male and female active SW faced economic strain during the pandemic, which led them to take greater risks, such as accepting new clientele, and therefore enduring more violence, as well as suffering from police violence (Adebisi et al., 2020; Boyer, 2020; Campbell et al., 2020; Kimani et al., 2020; Lam, 2020; Macharia et al., 2021; Olaya-Saldarriaga, 2021; Santos et al., 2021; Singer et al., 2020).

It is worth mentioning that SW have, in general, high-risk employment, which, in a conventional pre-pandemic world, exposes them to higher rates of victimisation than those of the general population. For example, Deering et al. (2014) conducted a systematic review (*N* = 41) and reported that SW’ lifetime prevalence of workplace violence varied from 45 to 75%, whilst their previous-year prevalence of workplace violence varied from 32 to 55%. Moreover, amongst the studies that have corroborated that SW have the highest risk of being victims of homicide, Potterat et al. (2004) indicated that violence and drug abuse were the primary causes of death amongst SW. Although there are no specific data on SW, the use of substances, particularly during the exercise of sex work, may be a risk factor for SW’ victimisation, as has been found previously for other populations, such as drug-addicted people (Aebi, 2006), homosexual men (Chakrapani et al., 2019), and adolescents (Morojele & Brook, 2006). It is particularly interesting to investigate this in Switzerland, as SW’ rates of drug consumption, although higher than those of the general population, are rather low nonetheless (Lociciro et al., 2017; Molnar et al., 2021). These high rates of victimisation contrast strongly with the general population’s prevalence of victimisation. For instance, the latest version of the *International Crime Victims Surveys* (ICVS, van Dijk et al., 2007) reported that in 2003/2004, the lifetime prevalence of being a victim of any common crime was, on average, 16% and of assaults and threats 3.1%. A recent Swiss victimisation survey (Caneppele et al., 2019) found that 1.3% of people had been victims of a physical assault in the street between 2013 and 2018 and 0.6% in 2018. Nevertheless, no data on the rates of victimisation of SW in Switzerland are available, except for qualitative studies published to date (Földhazi, 2010) that suggest that even within a regulatory framework, sex work is a risky job, as it can be for other professionals, such as police officers, fire-fighters, and prison staff.

Thus, this paper addresses SW’ victimisation and use of drugs in Switzerland during 2020, the first year of the COVID-19 pandemic. To overcome the challenges of conducting research during a pandemic, we took advantage of two critical circumstances: (1) the prior research work on prostitution that we conducted for years in collaboration with an SW-aid organisation (e.g. Molnar & Pongelli, 2019; Molnar et al., 2021; Ros, 2021; Ros & Rullac, 2020), and (2) the low stringency of coronavirus-related measures applied in Switzerland. Nonetheless, it is worth mentioning that, because of the lack of former studies on SW’ victimisation in Switzerland, our research does not provide causal information about its effects related to the pandemic. Instead, it describes SW’ violence- and drug use-related situation since the COVID-19 lockdown that took place in Switzerland in March 2020. Nevertheless, SW’ drug consumption can be compared with that in former years, as previous studies have been conducted that show a rather low prevalence of the latter amongst SW (Lociciro et al., 2017; Molnar et al., 2021).

## Data and Methods

### Description of the Field

#### Swiss Legal Framework of Prostitution

Different legal frameworks regulate sex work worldwide (Danna, 2014). In the case of the Swiss Confederation, sex work is a legal activity that is managed locally at the cantonal level (*ProCoRe*, n.d.). At the federal level, the Swiss Criminal Code (*RS 311.0 Code Pénal Suisse Du 21 Décembre 1937*) forbids sexual exploitation and pimping, and hence, a third party’s encouragement of prostitution (art. 195). In general, prostitution is legal in Switzerland if the SW is 18 years old or older, uncoerced, and not supported by a third party. In addition, the SW must be either a Swiss national or an EU foreigner. In practice, Swiss SW can announce themselves directly as freelancers and pay their taxes under this regime, whilst SW citizens of EU-member states can request a work permit to exercise prostitution in Switzerland as freelancers—normally the *B permit*—for a maximum of five years, renewable with an indefinite permit if the person is able to make a living from her work and has no debts. Further, EU citizens who want to work in prostitution temporarily are able to apply for the so-called *Announce of 90 days* (Secrétariat d’État aux migrations, n.d.), under which they are allowed to have employment in Switzerland for 90 days annually and pay their taxes in their home country. Nonetheless, extra-EU nationals, such as Africans, Asians, and Latin Americans, cannot apply for sex work-related residence permits and therefore, cannot migrate to Switzerland to work legally in prostitution.

In the canton studied here—the canton of Vaud—sex work is regulated by Law 943.05 on the exercise of prostitution from 2004 (Loi 943.05 sur l’exercice de la prostitution (LPros), 2004; Règlement 943.05.1 d’application de la Loi du 30 mars 2004 sur l’exercice de la prostitution (RLPros)). In this canton, sex work is permitted either in a delimited street perimeter in the city of Lausanne, capital of this canton or in the so-called erotic massage salons, which are apartments, clubs, or bars with an administrative authorization to rent rooms for tariffed sexual or erotic services. In the canton’s capital, it is not sex work per se that is allowed, but street soliciting (Ville de Lausanne, 2016), in which SW can solicit customers from 10 PM to 5 AM daily. However, sexual services are illegal in public spaces and prostitution outside of those places and times is subject to a fine by the Swiss criminal code (art. 199). SW who solicit in the street may go with the customer legally to the sex worker’s erotic massage salon, a hotel or the customer’s residence, whilst performing the sexual service in the street, a car, or at their own domicile is illegal.

The SW population is diverse and heterogeneous, and hence, it is challenging to estimate the number of SW. In 2009, Bugnon et al. (2009) estimated that 64% of SW worked in erotic massage salons, whilst the remainder worked either in the street, bars, or strip clubs. Escorts were the least prevalent—only 2% of SW appeared to be working in this domain, but this might be an underestimation because of the business’s covert nature. In 2015, it was estimated that, on an average day, there were 43 SW in the streets and 31 in erotic massage salons throughout Vaud (Biberstein & Killias, 2015). These figures, which are based upon data from NGOs, must be considered a proxy because of the high flux of SW entering and leaving Switzerland and the fact that some of them—in particular those who are undocumented migrants—do not announce their activity to the police.

During the pandemic, all forms of face-to-face sex work were forbidden in the canton from the 16^th^ of March until the 6th of June 2020. After that date, prostitution activities resumed, although the erotic massage salons stayed open only during the day until February 2021. Data from the NGO Fleur de Pavé (see above) indicate that, compared to 2019, the number of SW in the street decreased by 43% and in the erotic massage salons by 29% during 2020, but the SW who requested administrative assistance increased by 90% (Fleur de Pavé, 2020).

#### Gatekeeper

The gatekeeper of this research is the NGO Fleur de Pavé, an association that has been active in supporting SW and minimising the risks related to sex work for 25 years. Public authorities, i.e. the *Federal Office of Public Health*, the *Canton of Vaud*, and the *City of Lausanne*, subsidise Fleur de Pavé and private donors finance it as well. The association, which is composed of both social work-related professionals and former SW, works during weekdays in three shifts and places:

1. *In their office*, which is situated in the prostitution neighbourhood and open in the afternoons. The social workers offer administrative assistance and orientation to the SW on site or by e-mail or telephone
2. *In the erotic massage salons*, which the social workers visit in the afternoons to distribute prophylactic material and offer the SW advice and assistance
3. In the *prostitution area* at night, where the social workers stop with an auto-caravan and offer beverages and snacks, prophylactic material, and counselling.

The association’s work is guided by what is referred to as *low threshold social work*, a principle that ensures unconditional assistance to SW such that the counselling and aid provided depend solely upon SW’ needs, wishes, and priorities. During the pandemic, the SW who contacted the association did so to request all forms of assistance, from administrative aid to help applying for social aid or unemployment assistance and to humanitarian help in providing food or food vouchers because of the SW’ inability to work and lack of other economic resources, such as other employment or savings from their prior work (Ros & Molnar, in press).

In this context, the two researchers, a former Fleur de Pavé’s social worker and a researcher who was collaborating with the association already in another research project, proposed to the NGO to document the SW’ situation during the pandemic quantitatively. Therefore, we went out in the field and accompanied the social workers during their daily duties in the office, the erotic massage salons, and the prostitution perimeter, in the afternoons and nights. Many of the SW interviewed were acquainted with us already and the NGO’s social workers reassured those who were not of our trustworthiness. We go into further detail in the following subsections.

### Instrument for Data Collection

To collect the data systematically and obtain a general view of the pandemic’s effects on the SW’ lives, we developed a short questionnaire^1^ that explores various aspects^2^ during the first year of the COVID-19 pandemic: (1) SW’ physical and psychological health; (2) their view of the pandemic’s effects on their lives; (3) their access to financial and material resources; (4) their social rapport during the lockdown and the remainder of the year; (5) whether they worked during the period that sex work was prohibited and under which conditions; (6) whether they worked during the rest of the year and in which settings; (7) their needs; (8) their substance use during the first year of the pandemic compared to the prior period; and (9) their victimisation during 2020. This article addresses the last two variables, SW’ substance use and victimisation, in depth. With respect to SW’ substance use, the questionnaire addressed three drugs, both legal and illegal: (1) tobacco, (2) alcohol, and (3) illegal drugs. For each, the SW who used any of these substances could choose between six options that ranged from “During the pandemic, I consumed [much less/less/neither more nor less/more/much more] than before the pandemic.” We chose both legal and illegal substances because, regardless of their legal status, the World Health Organisation (n.d.) considers them drugs and therefore, they could have an effect on SW’ work, risk assessment, and use.

With respect to SW’ victimisation since March 2020, the questionnaire addressed five types of acts—both deviant and criminal—which we identified as the most prevalent in other research projects in Vaud as well as in the field: (1) theft; (2) physical assault; (3) sexual assault; (4) fraud by means of making the SW book an appointment and go to the client’s (who never showed up), and (5) fraud by means of tricks and false promises to pay after the sexual service. Whilst options 1–3 and 5 are illegal from a juridical point of view, we are aware that the no-shows are only deviant; nonetheless, we preferred to include them because they cause much patrimonial loss to the SW.^3^ If the SW answered affirmatively, in the same manner as in traditional victimisation surveys (see, e.g. van Dijk et al., 2007), detailed questions followed about the number of incidents suffered, the place and time of the victimisation, as well as whether the SW reported the event to the police.

We translated the questionnaire, which was developed originally in French, into Spanish, Romanian, and English, which are the main languages SW who work in the canton speak. Both members of the research team are fluent in French and English and the first author is fluent in Romanian and Spanish as well and therefore, nearly all questionnaires were administered in the SW’ language. Twenty questionnaires were administered in French, nine in Romanian, four in Spanish, and seven in English.

### Fieldwork and Data Collection

We included participants in our sample with diverse profiles who worked in different places and solicited customers both offline and online. We collected data from three sources: (1) 50 h of field observations in Lausanne’s prostitution neighbourhood, the office of the association, and erotic massage salons in Vaud, during which we conducted questionnaires and accompanied social workers in their daily tasks; (2) thirty-three face-to-face questionnaires; and (3) seven online self-administered questionnaires.

The 50 h that we spent in the field conducting surveys and accompanying the social workers allowed us to contextualise the pandemic-related settings of sex work in Vaud better. During our observations, we observed and conversed with both sexual and social workers. We went in the field 17 times to collect data and after each, we followed a methodological protocol in which we indicated the place where we conducted the observations and questionnaires, in which languages, with how many SW, and salient elements from the field work, such as methodological and ethical challenges associated, as well as relevant interactions with SW and social workers.

As stated, we also collected 40 SW’ responses to our questionnaire, whilst we surveyed 33 SW face to face during our field observations. Our response rate in the field was rather high and nearly all SW whom we asked to participate in an interview with us accepted. In addition, to reach other SW who did not necessarily work in official places, we disseminated the questionnaire as well on erotic advertisement websites in Vaud. In total, we messaged 84 cisgender women, seven transgender women, and 33 cisgender men, explained the goals and conditions of the study to them, and provided them the link to the online questionnaire, most often in their mother tongue. Seven SW to whom we sent the link to the online version of the questionnaire, one of whom we had met in person already, answered it online. The low response rate to the online questionnaire may have been attributable to the online profiles’ inactivity as well as to the SW’ mistrust of responding to an online survey.

With respect to the data collection techniques used with both samples, for the face-to-face interviews, we followed either *Computer-Assisted Personal Interviewing* (CAPI) or *Paper and Pencil Personal Interviewing* (PAPI), whilst for the online questionnaires, we followed *Computer-Assisted Self-Interviewing* (CASI). With respect to the CAPI method, we filled in the SW’ responses in front of them on our smartphone, which was connected online to our questionnaire. On other occasions, we also used PAPI by writing the SW’ responses on a printed version of the questionnaire and incorporated them later into the online questionnaire. For the online questionnaires, we created four additional questionnaires in the languages aforementioned and provided the SW their URL. SW were informed either orally or via the front page of the online questionnaire about the goals of the study and the conditions of participation: Not a remunerated study, voluntary basis, and right to withdraw at any time without explanation. With respect to the SW who completed the online questionnaire, we assumed their agreement to participate if the questionnaire was filled out nearly completely and submitted. Therefore, we discarded partial questionnaires that were not submitted ultimately.

Generally, face-to-face surveys were conducted individually with the SW either in the NGO’s office, on the street, or in unoccupied rooms in the erotic massage salons. Nonetheless, in five cases, we needed to depend upon third parties to conduct the interview. In four cases, the survey was conducted in the company of a social worker from the association who spoke other languages than those in which the team was fluent (e.g. Portuguese) and in one instance, a friend and roommate of the SW who stayed during the interview conducted the survey in an erotic massage salon.

### Data Analysis

We performed descriptive analyses, including the frequencies of the variables, as well as crosstabs, to assess the distribution of the sample with respect to victimisation and drug use. No statistical tests were performed because of the lack of sufficient observations. To compare groups, we presented frequencies and percentages as well. Nonetheless, because of the low frequencies, the percentages should be interpreted only for exploratory and comparative purposes to assess the differences and similarities between the groups better.

With respect to victimisation, we also calculated the *previous-year prevalence*, *previous-year incidence*, and *previous-year lambda variety*. The previous-year prevalence of victimisation refers to the percentage of people within a sample who have been a victim of n crime in the 12 previous months (Aebi, 2006). It can be expressed according to a specific offence or a group of offences. In this study, we addressed both. We present percentages only for illustrative purposes and it is important to keep in mind the small sample size. The previous-year incidence of victimisation represents the mean number of incidents the victims endured (Aebi, 2006). This was calculated by asking the respondents how many times they were victims of a specific offence (e.g. theft). Then, we summed the number of incidents of the same type the victims reported and divided them by the number of victims of, in our example, theft. Last, the previous-year lambda variety of the victimisation refers to the range of different offences suffered in the previous-year (Aebi, 2006). For the latter, seven types of offences were considered: 1) theft; 2) physical assault; 3) sexual assault; 4, 5, and 6) three types of fraud; and 7) wrongful restraint. We calculated the lambda variety of the victimisation (Aebi, 2006) as the sum of the range of offences each participant reported divided by the number of victims.

All three indicators are important to investigate, respectively, (1) the extent of the victimisation, (2) its repetition, and (3) its multiplicity (Aebi, 2006). Our analysis is justified by the literature, which has shown that victimisation is a rare event and amongst the victims, there is an even smaller number that endures victimisation either repeatedly or in a multiple manner (Cusson, 2019; Farrell, 1992; Wemmers et al., 2018). Therefore, the analysis of repeated and multiple victims allowed us to identify SW who are in greater need of identification and intervention.

### Ethical and Methodological Considerations

Because of both SW’ difficult-to-reach character and the sensitive nature of the questions that we addressed in this research, several ethical and methodological aspects should be mentioned briefly. Firstly, prostitution/sex work has been a highly controversial topic in academia (see Benoit et al., 2019) with respect to one’s moral position vis-à-vis sex work itself. *Grosso modo*, the academic debate has been divided between the so-called neo-abolitionist scholars—who cannot conceive of prostitution as a job, but only as exploitation of women who, more or less consciously, are coerced, in a factual or symbolic manner—and the pro-right scholars—who recognise SW’ self-determined right to work in whatever manner they decide and who denounce only sexual exploitation, but not free prostitution. In this sense, we did not begin our research with any axiom with respect to SW’ freedom or lack of freedom in general, nor the desirability of sex work in general because we believe that our research does not require any prior position.

In practice, we were able to gather data from 40 SW in three months because of our previous knowledge of the context and of many SW. Although this sample size would be considered small in other settings, during a pandemic and with a difficult-to-reach population, the members of which are highly reluctant to discuss issues with scholars seem a rather acceptable number in our view (see by comparison other studies conducted with SW during the COVID-19 outbreak that included from three to 30 SW in their samples). Further, we believe that interviewing in four languages in which both the SW and researchers are fluent—French, English, Spanish, and Romanian—increased the validity of the data collected, in the sense that the SW understood the terms used in the questionnaire more precisely than if they were explained only in French. This also diversified our sample, because there are many SW who come to Switzerland only occasionally and therefore, do not speak any other language than their own. In addition, we protected our participants by anonymising our data and providing herein only trends and general distributions of their drug use and victimisation and not any personal details about them when analysing specific variables. In addition, we disclose that we decided to retain categories that included less than five SW because they could not be identified. Similarly, we did not share any of the specific answers to the questionnaire with the NGO and stored our data in a safe Swiss server our universities provided.

We faced methodological and ethical challenges as well. First, we encountered logistic issues attributable to the COVID prevention measure of social distancing, such as the need to conduct surveys 1.5 m from the SW. As well, because of the pandemic, the member of the team who was in the prostitution area needed to stay out of the NGO’s auto-caravan and therefore, had to carry out the interviews in the open in the middle of the Swiss winter. Although the NGO provided a car at her disposal where she could warm up and conduct surveys, it was impossible to do so because many SW were not wearing face masks, which would have endangered her health. Moreover, in the street and the erotic massage salons, many SW were not wearing a face mask, which made us quite fearful and could have influenced our non-verbal reactions and thereby influenced the participants unconsciously. As well, the fact that several surveys were not conducted with the SW individually might affect the answers’ reliability; nonetheless, we are aware that this is a challenge even in high-standard European surveys, such as the EU-MIDIS study of the European Union Agency for Fundamental Rights (2017).

Addressing questions with respect to precariousness and victimisation at such a difficult time for many SW was often both ethically and emotionally challenging for us. To avoid having SW disclose difficult experiences that might trigger a revival of trauma, we always referred them to the NGO’s social workers at the end of the survey and explained to them that they could assist them further if needed. Similarly, interviewing people who shared with us that they had been, for instance, starving for several days, that they had lost their housing in the middle of the pandemic, or that they had been assaulted, obviously was highly challenging emotionally. To face that challenge and maintain relative neutrality, the research team also debriefed together, if needed, about the difficult situations lived in the field.

Last, one of the methodological challenges we faced was explaining the objective, purpose, and utility of our research to the SW. For example, two of them assumed, a priori, that we were conducting the study because we believe that SW have been vectors of coronavirus. Despite our explanations, they were still reluctant to participate and therefore, we did not conduct any surveys with those individuals.

### Participants’ Sociodemographic Characteristics and COVID-19-Related Situation

Table 1 illustrates the sociodemographic data of the 40 participants. Our sample was composed largely of cisgender women, who are in their 30 s on average, foreigners, and largely in an illegal situation. As well, the majority are single, but have children to look after. Most of the SW stated that the pandemic has affected them (very) adversely. This was stressed many times during our observations, and we even found SW who had not eaten for one or two days or who did not know where they would sleep the next day. Although these aspects with respect to precariousness and psychosocial effects during the pandemic are essential, they are not addressed in this article because they are the object of other publication.

**Table 1:** Sociodemographic and Covid-19-related data (*N* = 40)

Biological sex	38 Women 2 Men
Gender	39 Women 1 Non-binary
Age	Mean = 35.8; Median = 34; SD = 10.9; Min = 20; Max = 60
First Nationality	Brazilian = 3; Bulgarian = 1; Camerounian = 6; Colombian = 1; Dominican = 2; Ecuadorian = 1; Spanish = 3; French = 3; Nigerian = 5; Romanian = 12; Swiss = 1 *2 missing values
Legal situation	Legal situation (Swiss, residence permit or 90 days announce) = 15 Illegal situation = 20 *5 missing values
Civil status	Married/in partnership = 12 Single = 26 *2 missing values
Offspring	Yes = 22 No = 18
Effect of the pandemic	Very negative or negative = 33 Neither negative nor positive = 5 Positive = 2
Worked when sex work was prohibited (March–June 2020)	Yes, face to face = 10 Yes, webcam = 1 Yes, erotic phone = 1 No = 26

During the period that sex work was prohibited in the canton of Vaud (mid-March until the 6^th^ of June 2020), a quarter of the sample, all persons in an illegal situation, indicated that they have continued their work face to face.

## Findings

### Victimisation: Prevalence, Incidence, Variety, and Reporting to the Police

Overall, 17 SW (over 33 persons, 7 missing values) were victims of at least one of the following seven offences: theft; physical assault; sexual assault; three types of fraud; and wrongful restraint by a customer who forced the SW to stay in his car. For 33 SW, the previous-year prevalence of victimisation was 51.5%, although if we assume that the 7 SW who did not answer these questions were not victimised, the prevalence during the previous year would be 42.5%. In this section, we address the previous-year prevalence, incidence, variety, and reporting of victimisation (Table 2).

**Table 2:** Victimisation of SW (*n* = 33)

Types (multiple choice)	Prevalence (*n*)	Incidence (*n*)	Complaint (*n*)
Theft	11	1	1
Fraud 1 (displacement)	6	2.6	0
Fraud 2 (client does not pay for the service)	7	1.6	1
Physical assault	4	1	1
Sexual assault	4	1	1
Other: fake bank notes	1	1	0
Other: wrongful restraint	1	1	0
*Lambda variety* = *2*			

With respect to the incidence of victimisation in the previous year, during the first year of the pandemic, on average, SW were victims of customer fraud 2.6 and 1.6 times, respectively, whilst they were victims of one offence on average for the remainder of the crimes. Therefore, they were not victims of the same offence repeatedly, except for the customer’s fraud. This is a positive indicator, in the sense that they did not suffer continuous victimisations and although we do not have sufficient data to support this, it may indicate that, once victimised, they may have engaged in strategies to lower their risks of victimisation, for example, by asking for friends’ help or avoiding a certain type of customer. The lambda variety of victimisation was 2: each person endured two different offences on average. Eight SW were victims of only one of the offences included, whilst nine were victims of more than one. Specifically, two SW were victims of two different offences, six were victims of three offences, and one suffered four different offences.

SW tend not to contact the authorities when they are victims of a crime. Table 2 shows that one SW reported a theft to the police and that amongst the four victims of physical and sexual assault, as well as fraud, only one complained to the police. In addition, none contacted the police when they were victims of fraud (client dropping the SW), when they were forced to stay with the client in his car, or when they were given a forged banknote. With respect to the latter case, the SW reported that because she knew that she could be in trouble if found with a counterfeit banknote, she tore up and discarded the note. From our fieldwork, we can affirm that the reasons for not contacting the police are related primarily to the illegal status of the SW, who want to stay off of the authorities’ radar so they will not be expelled from Switzerland but also because they believe that the police cannot do very much to help them.

### Victimisation: Locations and Times

More participants (*n* = 11) reported that they had been victims of theft. Eight SW indicated that the thefts occurred in the street, two in the erotic massage salons, and one in a hotel. Vis-à-vis the times when these thefts occurred, nine SW said that they happened either in the evening or at night and only two indicated in the afternoon. From our field observations, it is plausible that these crimes are the most frequent because of the many opportunities that emerge for motivated offenders, particularly in the prostitution neighbourhood: The SW, who normally carry cash, are an attractive target for both customers and passers-by, who walk, and sometimes even loiter, in the zone for hours and who can monitor SW’ movements and decide the best moment to steal from them. As well, when they are with a customer, in his car, for example, SW need to leave their purse out of sight to engage in the sexual service or when working in an erotic massage salon, they sometimes go to the bathroom and leave their belongings with the customer.

Fraud is the second offence reported to have occurred most during the pandemic: Seven SW were victims of the deception of customers who promised falsely to pay for the service and six were contacted by clients who asked for an appointment at their place, but when the SW went to the meeting place, they were not there. These events took place in the street (*n* = 6), in an erotic massage salon (*n* = 2), at the SW’ residence (*n* = 1), at someone else’s (*n* = 1), i.e. the customer, and in other places as well (*n* = 2). Further, they occurred primarily during the evening (*n* = 4) and night (*n* = 5) and less often in the afternoon (*n* = 2) and morning (*n* = 2). As stated above, another form of fraud that a SW disclosed was being paid with a forged banknote.

Physical assaults (four victims) and sexual assaults (four victims) were less prevalent, although, in the entire sample, they still affected 10% of the participants. Physical assaults occurred exclusively in the street during the evening (*n* = 2) or night (*n* = 2). Sexual assaults took place primarily in the street (*n* = 3) and at the customer’s place (*n* = 1) at night as well. Another offence reported was the wrongful restraint of a SW whom her customer forced to remain in his locked car for a period of time.

### Drug Use During the Pandemic

Most SW in our sample do not use drugs in general (Table 3). With respect to prevalence, tobacco and alcohol are consumed most commonly (*n* = 15), whilst illegal drug use is an exception (*n* = 4), but still has a non-negligible prevalence (10%). Few SW used more substances during the first year of the coronavirus pandemic compared to the pre-pandemic period. Table 3 shows that seven SW indicated that they smoked more, five used more alcohol, and one consumed more illegal drugs (cannabis, cocaine, heroin, etc.) during the first year of COVID-19 than before the pandemic. The person who used more illegal drugs also used more alcohol and similarly, the SW who consumed fewer drugs also consumed less alcohol. With respect to the variety of products used, 14 SW do not use any of the three drugs studied and 20 used only one. Nonetheless, four SW use two substances and two use three.

**Table 3:** SW’ drug use during the first year of the pandemic compared to before the pandemic (*N* = 40)

	Tobacco	Alcohol	Illegal drugs
Does not consume	25	25	36
Consumed more during the pandemic	7	5	1
Consumed the same amount during the pandemic	8	7	2
Consumed less during the pandemic	0	3	1

*Variety*: 0 products = 14; 1 product = 20; 2 products = 4; 3 products = 2

### Characteristics of the Victims

Table 4 synthesises the differences between the victims (*n* = 17) and non-victims (*n* = 16) expressed in frequencies and percentages. The group of victimised SW was younger (mean age = 33.7 years old) than the SW who were not victims of a crime during 2020 (mean age = 36.9).^4^ As well, more people in the group of victims were in an illegal situation. The pandemic affected them slightly more as well and a greater proportion of them worked during the period that prostitution was prohibited. More victims worked in rather clandestine places during that time, such as their own residence and the customers’. This pattern still followed after prostitution was legalised again in June 2020, when more SW victims of a crime continued to work at the customers’ or at their own residence and less on the Internet or by telephone.

**Table 4:** Comparison between non-victims and victims

	Non-victims (*N* = 16)	Victims (*N* = 17)
*Age (average)*	36.9 (SD = 11.7)	33.7 (SD = 11)
*Sex*		
Cisgender woman	15	17
Transgender woman	1	
*Nationality*		
Swiss	1 (6%)	0
EU-member citizen	9 (56%)	7 (41%)
Extra-EU citizen	4 (25%)	10 (59%)
Illegal situation	5 (31%)	12 (71%)
*Pandemic: adverse effect*	12 (75%)	14 (82%)
*Worked during period when prostitution was prohibited (March-June 2020)*	3 (19%)	7 (41%)
Street	1 (6%)	4 (24%)
Salon	1 (6%)	1 (6%)
Internet	1 (6%)	0
Telephone	1 (6%)	1 (6%)
At own residence	0	3 (18%)
At the customer’s in the same city	1 (6%)	3 (18%)
At the customer’s in another city	2 (13%)	3 (18%)
*Worked since June 2020*		
Street	8 (50%)	8 (47%)
Salon	4 (25%)	6 (35%)
Internet	4 (25%)	1 (6%)
Telephone	2 (13%)	1 (6%)
At own place	1 (6%)	2 (12%)
At the customer’s in the same city	1 (6%)	8 (47%)
At the customer’s in another city	3 (19%)	5 (29%)
*Drug use*		
More tobacco than before the pandemic	4 (25%)	3 (18%)
More alcohol than before the pandemic	1 (6%)	3 (18%)
More illegal drugs than before the pandemic	0	1 (6%)
*Behaviour of their customers*		
Contacted during period when prostitution was prohibited	3 (19%)	10 (59%)
More customers insisted on a lower price	13 (81%)	12 (71%)
More customers insisted on having non-protected sex	6 (38%)	6 (35%)
Insisted on having sex without a face mask	6 (38%)	3 (18%)
Insisted that SW have a COVID-19 test	4 (25%)	4 (24%)

Vis-à-vis the victimised SW’ drug consumption, a higher percentage used more alcohol and illegal drugs during the pandemic than before that period, but a lower percentage reported that they consumed more tobacco. The results with respect to their customers’ behaviour are less straightforward: A greater proportion of victims were contacted by customers during the period that prostitution was prohibited, but, in general, a slightly higher percentage of non-victims reported that their clients insisted that they lower their price more than before the pandemic, as well as have unprotected sex. In addition, more non-victims had clients who insisted on engaging in the sexual service without a face mask, but at the same time, a greater proportion of non-victim SW’ clients insisted that they be tested for COVID-19.

### Victimisation of SW Who Worked During the Period That Prostitution Was Prohibited

We also attempted to determine whether there were differences between SW’ victimisation depending upon whether or not they worked when sex work was prohibited. The first finding is that indeed, as stated in the section above, 70% of the SW who worked when prostitution was prohibited from March to June 2020 were victims of at least one offence during 2020, compared to the 37% of those who did not work during the prohibition period. This was also corroborated by the variety (standard) of victimisations: SW who worked during the period that prostitution was prohibited were victims of 1.3 different offences, on average, whilst the number for those who did not work was 0.78.

Broken down by offences, we did not observe important differences between victims of fraud or theft. Nevertheless, 22.2% of the SW who worked during the prohibition were victims of sexual assault, compared to only 9.5% amongst those who did not work during the prohibition. Conversely, no SW who worked during the period that prostitution was prohibited was a victim of physical assault, compared to the 19% of victims amongst the group of those who did not work. Last, interestingly, 55.6% of the SW who worked during the prohibition were victims of no-shows on the part of their customers, compared to the 4.8% of the SW victims amongst those who did not work. Amongst the victims who did work during the period that prostitution was prohibited, none of them reported their victimisation to the police.

## Discussion and Conclusion

This paper shed light on the experiences and situation of 40 SW during the first year of the coronavirus pandemic in Switzerland, one of the few countries in the world where prostitution is legal. When compared to the general population, our participants’ victimisation (e.g. 10% for physical assault) is definitely higher than that of the general Swiss population (0.6% for the same offence) (Caneppele et al., 2019). Although we do not possess a Swiss comparison point vis-à-vis the victimisation of SW in a conventional setting—and therefore cannot carry out a pre-post-pandemic assessment such as the one Boyer (2020) conducted—our results are similar to those of previous studies, particularly with respect to the high prevalence of work-related victimisation in prostitution (Deering et al., 2014). Nonetheless, our study finds a nuance potentially related to the legality of sex work in Switzerland, with respect to the higher prevalence of property crime—i.e. theft and fraud—rather than violent offences during the first year of the pandemic. Nevertheless, their work conditions worsened during the pandemic. A number of the SW continued their work during the lockdown and similar to Azam et al. (2021), Adebisi et al. (2020), Kimani et al. (2020), and Macharia et al.’s (2021) studies, under more clandestine conditions, such as working at their residence and at the customer’s domicile. Further, in the same sense as Lam’s (2020) study, the migrant SW in an illegal situation could not stop working during the prostitution ban and thus exposed themselves to higher risks for victimisation. This is related to their illegal status, because of which they could not request state assistance that is provided to freelancers in Switzerland. Our findings are consistent as well with Singer et al.’s (2020) results with respect to the worsening of the customers’ behaviour during the pandemic, although in our study, only some of the behaviours worsened, such as the pressures to lower the prices as well as have unprotected intercourse. Another important finding that is likely also related to the legal status of sex work in Switzerland is that none of the participants mentioned any assault on the part of police officers. This contrasts strongly with the situation described in Campbell et al.’s (2020) study with respect to the brutal policing of Kenyan SW.

However, we cannot determine whether the SW’ victimisation increased or decreased because of the pandemic because of the lack of statistics with respect to their victimisation before the health crisis; with the arrival of the pandemic, we also observed that there were fewer SW, as well as fewer people walking through the prostitution neighbourhood at night, which makes it plausible that the lower informal social control may have facilitated some offences. Nonetheless, because unfortunately, we do not possess a comparative point of view, we cannot determine whether the number of motivated offenders decreased and therefore, fewer SW were victimised compared to the pre-pandemic period. In any case, we can affirm that non-documented migrants, young SW, and SW who needed to work during the COVID-related period when prostitution was prohibited were more at risk of suffering victimisation during 2020.

With respect to SW’ drug consumption, the results are consistent with previous Swiss studies of SW populations (Lociciro et al., 2017; Molnar et al., 2021): Although a small number of SW consume illegal drugs, most do not use products, such as cannabis or cocaine. However, many SW reported using more tobacco than before the pandemic, perhaps because of the strain imposed by the latter and the adverse effects it had on their lives and work.

In addition to those mentioned in the data and methods, a limitation of the study is that in contrast to Callander et al. (2021), we could not reach male SW, which leaves the question of the way the pandemic affected this population unanswered. The same is applicable to transgender SW, as only two participants identified themselves as such. Nonetheless, it is possible that the underrepresentation of transgender SW in our sample is attributable to the actual small number of them working currently in Vaud: during the recruitment process, we only encountered a total population of eight transgender SW. As stated in the Data and Methods section, amongst those, one was reached face to face and seven were contacted via their online advertisement.

The lack of a point of comparison for SW’ victimisation deserves further consideration. Therefore, we recommend conducting periodic victim surveys based upon the ICVS (van Dijk et al., 2007), for instance, of a considerable sample of SW in Switzerland. This could be useful to identify the extent of their victimisation, its specific forms, and emergent challenges. Prostitution is a rapidly changing phenomenon in Switzerland (Biberstein & Killias, 2015) and the challenges that existed ten years ago—for example, the high prevalence of drug-addicted SW—are seldom encountered today (Lociciro et al., 2017; Molnar et al., 2021). This is the reason why a periodic victim survey at the Swiss level could be of use to target the type of interventions better, as well as design better crime prevention programmes in sex work.

Many of the crimes the SW reported occurred in the street during the night. Therefore, in practice, it could be useful to apply basic situational prevention techniques (Cornish & Clarke, 2003), such as installing locker rooms in the street and increasing the cohesion amongst SW, which could increase the guardianship amongst them (in the same sense as Cohen & Felson, 1979) and thereby decrease their victimisation. In addition, it would also be important to revisit and identify safer and less expensive indoor locations that more SW could afford. For example, currently, a room in an erotic massage salon costs approximately 100 Swiss Francs daily (approximately 90 euros) or 3,000 Swiss Francs monthly (approximately 2,700 euros), a sum that many of the SW cannot afford. Further, crime prevention campaigns should continue to be conducted that inform SW about the risks of performing sex work at the customer’s abode, as well as the prevention techniques that they could apply to stay safe even in these settings (Molnar & Pongelli, 2019). Further, actions with respect to reporting victimisation to the police, the way to complain, as well as victims’ rights and assistance appear to be fundamental to encourage SW to report their victimisation to the police, which they do very infrequently at present.

Last, working under a prostitution ban, as well as without following the legal procedures, is associated with higher risks of victimisation. To address this, we recommend that researchers investigate citizens’ of the European Union reasons for working illegally in prostitution. However, it is not possible for extra-European nationals to work legally and therefore, in the event of another pandemic, if social and migration policies are not revisited, the outcomes of the risks described herein will be similar.

## Footnotes

1 The questionnaires are available in open access https://doi.org/10.5281/zenodo.5881464.

2 We designed the questionnaire in accordance with the NGO’s orientations with respect to the most relevant and challenging aspects the SW faced during the pandemic, as well as according to other surveys, such as the ICVS (van Dijk et al., 2007) and former research on sex work.

3 For instance, in other jobs, e. g. the medical field, if the customer does not come to the appointment, the professional still charges the client with the fee; this is not the case in prostitution.

4 The group of multi-victimised SW was the youngest (mean age = 28 years old), but there were no clear age differences between the non-victims and the victims of 1–2 offences (mean age 37 and 38 years old).

## Declarations

### Availability of data and material

Project questionnaires are available at https://doi.org/10.5281/zenodo.5881464.
